# Nutrition in Pediatric Metabolic Dysfunction–Associated Steatotic Liver Disease (MASLD)

**DOI:** 10.1155/jnme/3692044

**Published:** 2026-08-17

**Authors:** Dieudonne Nonga, Jonathan Gisser, Eileen Chaves, Angel DiPangrazio, April Lehman, Sindhu Pandurangi, Sara Hassan, Christopher Chu, Alvaro Flores

**Affiliations:** ^1^ Department of Pediatrics, Division of Gastroenterology, Hepatology and Nutrition, Nationwide Children’s Hospital, The Ohio State University, Columbus, Ohio, USA, osu.edu; ^2^ Department of Pediatrics, Division of Pediatric Psychology and Neuropsychology, Nationwide Children’s Hospital, The Ohio State University, Columbus, Ohio, USA, osu.edu; ^3^ Center for Healthy Weight and Nutrition, Nationwide Children’s Hospital, Columbus, Ohio, USA, nationwidechildrens.org; ^4^ Division of Genetic and Genomic Medicine, Nationwide Children’s Hospital, Columbus, Ohio, USA, nationwidechildrens.org; ^5^ Department of Pediatrics, The Ohio State University, Columbus, Ohio, USA, osu.edu; ^6^ Department of Pediatrics, Division of Gastroenterology, Hepatology and Nutrition, Children’s Medical Center, University of Texas at Southwestern, Dallas, Texas, USA; ^7^ Department of Pediatrics, Division of Gastroenterology, Hepatology and Nutrition, Rady Children’s Hospital, University of California San Diego College of Medicine, San Diego, California, USA, rchsd.org; ^8^ Department of Pediatrics, Division of Gastroenterology, Hepatology and Nutrition, Children’s Nebraska, University of Nebraska Medical Center, Omaha, Nebraska, USA, unmc.edu

**Keywords:** disordered eating, eating disorder, food access and distribution, malnutrition, monitoring, nutrient deficiencies, nutrition, pediatric MASLD, screening

## Abstract

Lifestyle change, including weight loss in children with overweight and obesity, through intensive health behavior and lifestyle treatment (IHBLT) is one of the main pillars in the management of pediatric metabolic dysfunction–associated steatotic liver disease (MASLD). Approximately 10% of school‐aged children in the United States—and an even higher percentage of children with obesity—are affected by MASLD. The stigma, combined with peer and society pressure to maintain a “healthy” weight, as it pertains to MASLD, has led a percentage of affected children to adapt restrictive patterns of eating that have culminated in malnutrition, nutrient deficiencies, disordered eating, anxiety and mood, and eating disorders. Prompt recognition through screening and targeted interventions is needed to address this crisis. Our paper offers a concise review and approach to the nutrition evaluation and assessment of malnutrition and nutrition deficiencies in children with pediatric obesity and MASLD and addresses interventions considered to mitigate this health outcome. We reviewed the literature pertaining to pediatric MASLD and malnutrition, nutrient deficiencies, eating behaviors, eating disorders, disordered eating, and food insecurity in the pediatric population. We focused on the evaluation, assessment, and management of malnutrition and nutrition and behavioral interventions recommended in pediatric MASLD. Overall, IHBLT alone has not been sufficient to curb the pediatric obesity and MASLD epidemic, and there has been a growing interest in specific pediatric MASLD pharmacotherapies and their related nutritional and behavioral side effects. Ultimately, assessing and addressing nutrient deficiencies, mood disturbances, and disordered eating behaviors is critical to mitigating the long‐term consequences of this disease, while improving food access and distribution can further optimize metabolic health outcomes through high‐quality nutrition.

## 1. Introduction

Consumption of low‐nutrient foods with added sugar and excess fat plays a role in the prevalence of malnutrition and nutritional deficiencies, in general, and pediatric metabolic dysfunction‐associated steatotic liver disease (MASLD) and steatohepatitis (MASH), in particular. Approximately 10% of children in the United States and 1 out 4 of children with obesity are affected by MASLD [1]. Intensive health behavior and lifestyle treatment (IHBLT), centering around the combination of healthy eating habits and exercise, remains the foundation for MASLD and MASH management. More than a third of pediatric MASLD patients progress to MASH despite IHBLT, and close to a quarter have advanced fibrosis [2]. The progression and rising incidence of MASLD is influenced by multiple factors including the following: economically imbalanced food distribution and limited access to high‐quality food and healthcare; lack of MASLD‐targeted pharmacotherapies and multicenter pediatric clinical trials to test these therapies; the absence of public health regulatory policies necessary to address these barriers. Ensuring adequate distribution and access to high‐quality food and healthcare can enhance the impact of IHBLT. Pharmacotherapies targeting individual MASLD risk factors, especially obesity, have been one approach to augment the efficacy of IHBLT; however, they carry their own inherent risks.

The stigma associated with obesity and being overweight has raised concerns about eating and mood disorders in children with obesity. Aggressive lifestyle interventions have contributed to the emergence of eating disorders (EDs) in this population. This makes eating habits, diet, behavior around eating, and mood assessment a cornerstone to the evaluation of MASLD in children with obesity. Screening for nutrient deficiencies associated with high‐calorie and low‐nutrient intake, often by a resolute registered dietitian, is necessary. In this article, we will review the nutrition assessment of pediatric patients with MASLD and MASH, cover the most common nutrient deficiencies in pediatric MASLD, touch upon monogenic metabolic defects impacted by nutrition that may lead to hepatic steatosis, and elaborate on the impact of reinforcing lifestyle changes on psychosocial behaviors, as it pertains to EDs. Finally, we offer some insights on improving health outcomes through optimizing distribution and access to high quality food.

### 1.1. Nutrition Assessment in MASLD

A thorough nutrition assessment is necessary to appropriately counsel and educate caregivers to prevent progression of pediatric MASLD. This includes assessing food and nutrient intake, anthropometric measures, growth patterns, presence of comorbidities, socioeconomic status, obesogenic and weight‐loss pharmacotherapies, biochemical data, mental health status, and level of family involvement. Obtaining a dietary recall to identify staple foods is an important first step to assessing nutrient intake [3]. Eating behaviors, such as hyperphagia, compulsive eating, abnormal meal spacing, sneaking or hiding food, excessive snacking or grazing, and meal skipping, should be documented. Given the known role of sugar‐sweetened beverages (SSBs), fructose, and refined carbohydrates consumption in the pathophysiology of MASLD, intake of these products should be quantified.

Meal skipping and grazing or snacking are noteworthy behaviors. Skipping meals can lead to larger portion sizes later in the day and frequent grazing or snacking. It is important to have a regular meal and snack schedule at home for consistency. It is the caregiver’s role to choose when the child or family eats, what to eat, and where to eat. It is the child’s role to choose how much or whether they will eat what is offered [5]. If caregivers express concern for hiding or sneaking foods at home, a discussion around keeping these foods out of the home and replacing them with foods that are high in fiber and protein can be beneficial. Also, placement of food at home can be an effective strategy to encourage consumption of more nutrient‐dense foods. This can include keeping fruits, vegetables, and high‐protein snacks at the front of the refrigerator so they are easy to see and grab (See Table 1).

**TABLE 1:** Nutrition assessment for MASLD [3, 4].

Obesity BMI ≥ 95th percentile
Comorbidities (Type 2 diabetes, dyslipidemia, hypertension)
Anthropometric measures and growth patterns/trends
Biochemical data and medical tests—alanine amino transferase (ALT)
Readiness for change
Pharmacotherapy
Level of family involvement
Socioeconomic status (SES)
Social determinants of health (SDOH)
Food and nutrient intake
SSB intake and frequency
Meal and snack patterns
Behaviors around food and eating (i.e., picky eating, sneaking, or hiding food)

*Note:* Adapted from the pediatric nutrition care manual.

Picky eating can also contribute to MASLD progression. There is currently no consensus on the definition of picky eating. Some common features include pathologic selectivity resulting in a severely restricted food repertoire, refusal of new foods (neophobia), and sensory aversion to smell, flavor, appearance, and/or texture of foods [6]. The net effect of these factors is the consumption of a diet rich in refined carbohydrates. As these products are poor in inducing long‐term satiety, the net result is often larger portion sizes and inadequate intake of fiber and protein, potentiating MASLD progression. Children may need to be referred for behavioral feeding support depending on the number of foods accepted and their response or behaviors around trying new foods [7].

In the pediatric population, an understanding of the parent–child dynamic is critical since dietary adjustments will often rely on caregiver temperament and capacity to implement changes. Table 2 lists questions to consider when assessing family and home‐food environment.

**TABLE 2:** Assessing family and home‐food environment and dietary recall [3, 8, 9].

Questions to consider for family dynamics and home‐food environment	Questions to consider for dietary recall
Who lives at home with the patient? (Mom, dad, caregiver/guardian? Are parents together or separate? If separated, how many days do they spend with each parent? Siblings? Do other family members or family friends help care for and feed the patient?)	What does a typical day of eating look like? At what times does the child eat during the day and what will they eat?

What foods are available at home?	Do they eat quickly? If so, how quickly do they eat and do they ask for second helpings of food soon after?

Who primarily cooks for the family and who grocery shops?	Is the child hiding and/or sneaking food at home? If so, consider asking the following:
	• What foods are they hiding or sneaking?
	If it is mostly energy dense foods (sweets, soda, chips, etc.), have caregivers attempted to not have these foods at home?

Is the family supportive of lifestyle changes or resistant? (e.g., unwilling to make these changes for the entire family and therefore singling out the patient)	Does the child have an early return to hunger after meals? If so, consider asking the following:
	• Are meals high in protein and fiber?
	• Do meals consist of 3–5 food groups each?
	Is the child skipping meals or snacking throughout the day?

Does the family have resource needs or concerns? (use of food pantries, SNAP benefits, or worry about not having enough food, food not lasting throughout the entire month)	Is the child a picky eater? If so, consider the following:
	• What foods are they selective of? And what are preferred foods?
	• How often are new or nonpreferred foods offered at home? Who offers these foods? What is the response when new or nonpreferred foods are offered?
	• Are caregivers or others in the home picky eaters or have a history of picky eating?
	• Is the child sensitive to textures or certain flavors?
	If the child accepts 5 foods or less, a referral for behavioral feeding support may be beneficial

Social determinants of health (SDOH) should be explored, as they underpin a family’s capacity to adhere to medical recommendations. Similarly, an inventory of potential barriers to implementing nutritional changes should be obtained in the initial assessment to appropriately tailor recommendations. Potential barriers include, but are not limited to, busy work and family schedules, multiple caregivers, financial and resource constraints, and insufficient family engagement.

#### 1.1.1. Nutrition Interventions for MASLD

A diet high in simple sugars is one of the major contributors to the development and progression of MASLD [10]. Fructose has been found to stimulate hepatic de novo lipogenesis that then leads to lipid accumulation in the liver [11]. SSBs, such as soda, juice, sweet tea, and lemonade, are often the primary source of sugar in children and are high in sucrose. Therefore, eliminating or reducing these beverages is one of the first recommended interventions; another intervention focus includes minimizing the consumption of refined carbohydrates, which are converted to sugar as well as foods that are high in saturated fat [12].

Nutrition interventions include education on the importance of fibers, nutrient‐dense foods, protein, eating meals consistently throughout the day, recognition of hunger and fullness cues, and education on age‐appropriate portion sizes. Common themes include an increased consumption of fruits, vegetables, whole grains, legumes, and lean protein, along with decreased intake of *SSBs*, ultraprocessed foods, and foods high in saturated fats [13–15]. Dietary approaches studied in the pediatric population are summarized in Table 3.

**TABLE 3:** Dietary considerations in the treatment in Pediatric MASLD.

Diet	Structure	Mechanism	Effect on micronutrients	Pros and cons
Carbohydrate reduced diet	Limit the percentage of total energy intake from carbohydrates (CHO)	**- Decreased insulin resistance** ‐ Decreased lipogenesis and hepatic fat storage ‐ Increased glycolysis and gluconeogenesis for energy production	‐ Vitamin D and trace element deficiency ‐ Dyslipidemia (increased total cholesterol and triglycerides) ‐ Osteopenia	Pros: limiting CHO often decreases intake of ultraprocessed foods; weight loss variable but seen more in very low CHO diet. Reported decrease in hepatic steatosis [13, 16, 17]. Cons: Lack of standard definition for low or very low CHO diets; frequently accompanied by increased dietary fat intake. May be harder to follow for children and lack of data to support long‐term use [16, 17].

Anti‐inflammatory and plant‐based diets	‐ Plant‐based protein sources (legumes, nuts, soy) ‐ High‐fiber intake ‐ Limitation of refined carbohydrates, dairy, and fats ‐ ± Avoidance of animal products ‐ Addition of anti‐inflammatory spices	‐ Anti‐inflammatory effects ‐ Antioxidant benefits ‐ Improved insulin sensitivity ‐ Microbiome modulation	‐ May be at risk for vitamin D, calcium, zinc, iron, and B12 deficiencies	Pros: lower protein costs than animal‐based products. Regression of MASLD on ultrasound and transient elastography, as well as decreasing trends of ALT, AST, fatty liver index, and MASLD [13, 25]. Cons: may require micronutrient monitoring and supplementation (vitamin D, calcium, B12, iron, zinc).

Mediterranean	‐ Limiting processed foods ‐ Intake of unrefined, fresh products ‐ Reduced consumption of saturated fat, red meat ‐ Total fat accounts for 35%–45% of dairy intake, with at least half coming from monounsaturated fats ‐ Increased intake of complex carbohydrates, plant polyphenols	‐ Improved insulin sensitivity ‐ Reduced oxidative stress ‐ Anti‐inflammatory effects ‐ Microbiome modulation ‐ Increased SCFA production	‐ Low prevalence of micronutrient deficiencies	Pros: high adherence, sustainability, reduction of lifetime personal costs, reduction in long term societal costs. Reported reduction in hepatic steatosis, liver enzymes, and insulin resistance, with no significant reduction in the energy required for growth in children with MASLD [15, 22].

Ketogenic diet	High fat (> 60%), low carbohydrate (∼8%), and moderate protein (∼30%) with ketosis	‐ Decreased insulin resistance ‐ Decreased lipogenesis and hepatic fat storage ‐ Increased glycolysis and gluconeogenesis for energy production ‐Increased free fatty acids and ketones decrease serum glucose and insulin	‐ Multivitamin supplementation recommended ‐ Vitamin D and trace element deficiency ‐ Dyslipidemia (increased total cholesterol and triglycerides) ‐ Osteopenia	Pros: improves glycemic control, used as therapy in other pediatric conditions; initial high adherence; significant weight loss possible Cons: high fat intake; reduced intake of grains, fruits, and vegetables with nutrient deficiency risk; food group restrictions make diet difficult to sustain. Risk of acute pancreatitis, kidney stones, and development of constipation. Lack of research and data to support long‐term use in pediatric obesity and MASLD population [18].

Time‐limited eating (aged 5 years and older) [19]	‐16 h/day fasting with 8 h eating window for 3–5 days per week	‐Improvement in insulin sensitivity and decreased lipotoxicity	‐ Ensure nutritional quality during feeding time to avoid deficiencies	Pros: families reported easy to implement across a variety of ages and cognitive abilities, flexibility with food choices and quantity, less rigid and requires less tracking Cons: eating windows may conflict with school, social, and family schedules; risk of nutrient deficiency; lack of research and data to support long‐term use, requires highly motivated and supportive caregivers.

Intermittent Energy Restriction (aged 12 years and older) [20]	‐Low‐energy diet 3 days/week and an eating plan consistent with national dietary guidelines 4 days/week	‐ Preferential use of fatty acids over glucose for energy ‐ Increased lipolysis, decreased lipogenesis, increased hepatic fatty acid oxidation.	‐Ensure nutritional quality during feeding time to avoid deficiencies	Pros: adolescents studied found this dietary pattern acceptable and flexible with school and social functions Cons: risk of malnutrition, lack of research and data to support long‐term use, concern for psychosocial well‐being in the long‐term, lack of research in pediatric patients with mood concerns.

*Note:* Adapted from Karjoo et al. [16].

There is no one‐size‐fits‐all specific diet that has been proven to be superior for the treatment of MASLD, and studied dietary interventions addressing pediatric metabolic risk factors have been applied to pediatric MASLD, but only very few have shown a sustainable impact on liver steatosis or aminotransferases in pediatric cohorts [16]. The Mediterranean and reduced carbohydrate diets have shown encouraging results [14, 17].

More restrictive diets such as ketogenic and intermittent fasting, if contemplated, should be considered with greater caution after a thorough and individualized assessment in the pediatric population and monitored judiciously, given their restrictive nature (Table 3) [16, 18].

Time‐limited eating (aged 5 years and older) and intermittent energy restriction (aged 12 years and older) have been evaluated in pediatric obesity populations but should be approached with caution [19]. In light of their restrictive nature, a thorough individualized assessment that includes screening for disordered eating behaviors (DEBs) and risk should be performed.

Calorie counting or restriction for weight management is discouraged in pediatric patients with MASLD, due to its association with risk for the development of EDs, disordered eating, negative impact on the enjoyment of food, and limited long‐term efficacy [20, 21]. It has been recently demonstrated that disease severity correlates more closely with high intake of sugars and carbohydrates, rather than caloric intake [17]. Therefore, interventions for pediatric MASLD should focus on nutrient quality rather than caloric quantity [13, 22, 23].

Prevention of disordered eating and progression to EDs, while providing nutritional intervention, is key for the success of nutritional therapy and optimal obesity‐related health outcomes including MASLD treatment. Efforts should be overtaken to favor behavior change and long‐term sustainability of this change. Interventions include use of nonstigmatizing language, avoidance of weight bias, weight‐neutral approaches where appropriate, varied nutrient‐rich rather than restrictive diets, and intense focus on weight goals [24]. Instead, treatment focus should be on helping families create realistic, doable goals focused on health behavior change that can be implemented in their lives. Focusing on patient‐centered health behavior goals allows families to tell healthcare providers what they feel is doable and to problem‐solve barriers to these goals in real time to increase implementation of these goals once they leave the clinic space [25].

### 1.2. Nutritional Deficiencies and End‐Organ Consequences in MASLD

Nutritional deficiencies are common in pediatric MASLD, encompassing vitamin D deficiency, deficits in essential micronutrients, and low fiber and protein intake.

Vitamin D deficiency is particularly prevalent and has been implicated in the pathogenesis of steatosis, inflammation, and fibrosis through its effects on insulin sensitivity, immune modulation, and hepatic lipid metabolism [26, 27]. Deficiencies in B vitamins, including niacin (B3) and cobalamin (B12), impair energy metabolism and contribute to hepatic fat accumulation [28]. Trace minerals are also affected: Zinc deficiency is linked with increased fibrosis and hepatic injury, magnesium deficiency impairs insulin sensitivity and glucose metabolism, and selenium deficiency reduces antioxidant capacity, with early studies suggesting that supplementation may slow liver disease progression [28].

Inadequate fiber intake contributes to gut dysbiosis and increased hepatic inflammation, which not only leads to liver injury but also disrupts metabolic balance more broadly [29]. In an individual with MASLD, this can compound disease burden and potentially accelerate the transition from simple steatosis to more advanced stages of liver injury.

Insufficient protein intake is frequently observed in this population, which can further exacerbate nutritional imbalance and increase the risk of sarcopenia, and evidence in adults and children indicates that it likely accelerates disease progression [30]. The combination of poor dietary quality and nutrient deficiencies manifests in significant clinical consequences. Sarcopenia, characterized by reduced muscle mass and strength, is increasingly recognized in pediatric MASLD, with prevalence estimates ranging from 20% to 40% [31, 32]. In adults this paradoxical coexistence of muscle loss and obesity, termed sarcopenic obesity, arises from shared mechanisms of chronic inflammation, insulin resistance, oxidative stress, and mitochondrial dysfunction, and in children with MASLD, it is strongly associated with more advanced fibrosis, greater risk of progression to steatohepatitis (MASH), and increased overall mortality [33, 34]. Although sarcopenic obesity is a very well‐established entity in the adult population, the definition is not yet well established in children, and further research is needed to understand these changes in the pediatric population. However, it is suggested that it could be beneficial to screen for sarcopenic obesity with body composition assessment in children with MASLD given the potential risks [30].

### 1.3. Screening for Micronutrient Deficiencies in MASLD

Data on micronutrient deficiencies screening and prevalence in MASLD are limited in the pediatric population but are well established in children with obesity, which represents the most common phenotype of MASLD. It is estimated that over 50% of patients with obesity have nutritional deficiencies, and 85% of bariatric patients have at least 1 micronutrient deficiency [35]. Although there are no specific guidelines for screening of micronutrient deficiencies in children with obesity, except for when undergoing bariatric surgery, it may be beneficial to consider other cost‐effective interventions such as supplementation of certain vitamins, such vitamin D, or use of multivitamin given the high risk of micronutrient deficiencies in this population [36]. It is also of great importance for the clinician to assess for micronutrient deficiencies during the clinical evaluation and provide recommendations for supplementation and treatment.

### 1.4. Nutrient Supplementation for Treatment of MASLD

In addition to vitamin and mineral deficiencies, pediatric patients with MASLD often lack other key nutrients, including antioxidants and polyunsaturated fatty acids (PUFAs) [23]. Antioxidants such as vitamin E reduce oxidative stress, while PUFAs—found in flaxseed, fish, and nuts—help inhibit de novo lipogenesis [23, 39]. Although vitamin E supplementation has been well studied, results have been mixed, and long‐term use is not currently recommended due to safety/toxicity concerns [40]. Trials of ginger supplementation have shown improvement in hepatic steatosis by ultrasound and reductions in ALT levels, likely related to its antioxidant and anti‐inflammatory properties [41]. Similarly, studies of DHA supplementation have demonstrated benefit, with a suggested significant decrease in hepatic fat without a dose‐related difference [42].

### 1.5. Genetics and Metabolic Factors in Hepatic Steatosis and MASLD

Genetic factors may affect the impact of carbohydrate and simple sugar intake on de novo hepatic lipogenesis. Individuals with variants in the patatin‐like phospholipase domain‐containing protein 3 (*PNPLA3*) gene experience increased hepatic fat deposition, in a dose‐dependent manner, with carbohydrate and simple sugar intake [43]. Therefore, carbohydrate‐restricted diets may be beneficial for those patients with pathogenic variants.

Several inherited metabolic and monogenic disorders, including tyrosinemia, galactosemia, hereditary fructose intolerance, glycogen storage diseases, fatty acid oxidation disorders, and urea cycle disorders, can lead to hepatocellular injury with steatosis, through the accumulation of toxic metabolites or deficiencies in critical compounds, and each requires a specific dietary treatment to prevent progression [44–52]. Strategies include restriction of the offending substrate (e.g., tyrosine, galactose, and fructose), supplementation with alternative energy sources (e.g., cornstarch and medium‐chain triglycerides), and targeted nutrient or pharmacologic therapy (e.g., nitisinone, nitrogen scavengers, and medicalized foods and formulas). Recognizing MASLD within this context is essential, as dietary therapy can dramatically alter outcomes, reduce progression to fibrosis or cirrhosis, and improve long‐term prognosis [53].

### 1.6. Impact of Reinforcing Lifestyle/Nutritional Changes on Social and Psychosocial Behaviors

IHBLT is the foundation for treatment of pediatric obesity and MASLD. This focuses on helping children, adolescents, and caregivers amend what and how they are eating and increase their involvement in physical activity. The American Gastroenterology Association recommends structured interventions combining dietary changes and increased movement, which help to improve liver histology, reduce hepatic fibrosis, and improve obesity, sarcopenia, and quality of life [28]. IHBLT involves engagement with families to provide support, patient‐centered goal setting related to nutrition and physical activity changes, assessment of barriers to making changes, problem‐solving, and face‐to‐face opportunities to practice behavior changes. The gold standard model of IHBLT delivery is via multidisciplinary care, including a pediatric health psychologist or behavioral medicine specialist (licensed professional counselor or social worker), dietician, physical activity specialist, and medical provider. Plans should be individualized to the pediatric patient and family’s social and cultural context. Family involvement in the creation of the plan is key to optimizing outcomes and improvements in pediatric MASLD; helping children, adolescents, and families make changes to what and how they are eating and moving often leads to positive changes in other areas of life, including positive impacts on peer relationships, school performance, and participation in social activities [54]. These domains are often impaired in children and teens with MASLD due to comorbidities of sleep apnea, mood disorders, and obesity [27, 55]. Developing family‐centered goals effecting changes in health behaviors in a culturally appropriate fashion is suggested to be the primary intervention for improving both medical and psychosocial outcomes in pediatric obesity and MASLD [56, 57].

### 1.7. EDs and Disordered Eating in MASLD

EDs and disordered eating are increasingly recognized as relevant and significant comorbidities in metabolic diseases and in pediatric MASLD [58]. Though often used interchangeably, they differ in clinical severity. Disordered eating describes a spectrum of unhealthy behaviors and attitudes toward food and body image that do not meet the diagnostic criteria for a clinical ED. EDs are severe illnesses that cause a significant, persistent impairment in eating‐related behaviors that negatively impact physical and mental health. In this review, DEBs will be used to describe the range of disorders, including subclinical and clinically significant presentations of EDs (Table 4).

**TABLE 4:** Micronutrient deficiencies in MASLD [37, 38].

Micronutrient deficiency	Mechanism in MASLD pathogenesis/progression	Effects on pediatric MASLD	Extrahepatic manifestations
Vitamin D	Impaired vitamin D receptor signaling increases hepatic lipid accumulation, inflammation, and insulin resistance; worsens muscle protein Synthesis and mitochondrial function	Increased hepatic steatosis, inflammation	Worsened sarcopenia and muscle weakness

Zinc	Impaired antioxidant enzyme function, altered protein synthesis, and immune dysregulation	Worsened hepatic inflammation	Impaired muscle protein synthesis, myopathy

Selenium	Reduced glutathione peroxidase activity increases oxidative stress and inflammation	Increased risk of liver fibrosis	Increased risk of myositis, muscle cramps

Magnesium	Impaired insulin signaling and mitochondrial dysfunction	Exacerbated insulin resistance, increased hepatic steatosis	Muscle fatigue

Vitamin B12	Impaired DNA synthesis and methylation, altered energy metabolism	/	Muscle weakness, neuropathy, worsened sarcopenia, anemia

Folate (vitamin B9)	Impaired DNA synthesis and methylation, increased homocysteine	/	Muscle weakness, macrocytic anemia, impaired muscle repair

Thiamine (vitamin B1)	Impaired carbohydrate metabolism, mitochondrial dysfunction	/	Muscle weakness, fatigue, risk of lactic acidosis, wet and dry beriberi, Wernicke–Korsakoff syndrome

Niacin (vitamin B3)	Impaired NAD/NADP‐dependent metabolic pathways	Impaired hepatic metabolism	Fatigue, muscle weakness, impaired muscle energy metabolism

Pyridoxine (vitamin B6)	Impaired amino acid metabolism, neurotransmitter synthesis	Impaired protein synthesis	Muscle weakness, neuropathy

Significant DEBs have been reported in adolescents with MASLD, MASH, and severe obesity [59]. These include eating in the absence of hunger, eating past the point of satiety, and meal skipping, which serve as barriers to weight loss, potentially perpetuating MASLD. Nocturnal eating, defined as the consumption of food after the evening meal and/or during the night after going to bed, also occurs more often in children and adolescents with MASLD and MASH [60]. This behavior is distinct from occasional late‐night snacking and is typically associated with disrupted circadian rhythms, increased caloric intake, and adverse metabolic outcomes [61].

It is important for medical providers and multidisciplinary MASLD team members to screen for DEBs prior to starting treatment for MASLD. Timely recognition of DEBs in children and adolescents with MASLD is crucial to achieve improved health and quality of life [62, 63]. Several screening tools exist to identify EDs, though none specifically target subclinical disordered eating (Table 5) [64].

**TABLE 5:** Pediatric eating disorder screeners [63, 64].

Screening tool	Population validated	Pros	Cons
SCOFF	≥ 11 years old	‐ Simple and quick to administer ‐ High sensitivity (100%) and specificity (87.5%) ‐ Useful for initial screening in various settings	‐ May not capture all types of eating disorders. ‐ Limited to older children and adolescents ‐ Developed before BED, AAN, and ARFID became recognized diagnoses

CHEAT	8–15 years old	‐ Specifically designed for children and adolescents ‐ Assesses a wide range of disordered eating behaviors ‐ High reliability and validity	‐ Requires more time to complete (26 items) ‐ May need professional interpretation

EDE‐Q	≥ 14 years old	‐ Comprehensive assessment of eating disorder symptoms ‐ Includes subscales for detailed analysis ‐ Adapted versions available for younger children	‐ Longer questionnaire (28 items) ‐ May be challenging for younger children to self‐report

ADO‐BED	12–18 years old with obesity	‐ Effective for identifying binge eating disorder in adolescents ‐ High sensitivity and specificity ‐ Useful for high‐risk populations	‐ Focused primarily on binge eating disorder ‐ Limited research on use with younger children

DEBs remain understudied, and given that it often represents a subclinical stage that can progress to a diagnosable ED, validated tools for early detection are urgently needed [65].

While IHBLT is the standard of care for the treatment of children and adolescents with MASLD, some of its components, such as restrictive calorie goals, meal tracking, limiting certain food groups (e.g., carbohydrates), or significantly increasing activity, can lead to harmful patterns such as food restriction, all‐or‐nothing thinking, elimination of entire food groups, or compensatory behavior such as excessive exercise [66]. These potential risks highlight the importance of careful clinical oversight and patient education.

Despite these concerns, the participation of children and adolescents in structured obesity treatment programs that promote regular meals, diverse nutritious foods, reduced *SSBs*, and joyful movement is associated with reduced ED prevalence, risk, and symptoms [67]. Should concerns arise for a diagnosable ED, a multidisciplinary team approach should be sought for ED specific treatment.

### 1.8. Improving Nutrition and Health Outcomes

Improving access to healthy foods, safe exercise, preventive healthcare, education, and mental health resources should be a priority to prevent and minimize the effect of obesity and MASLD. While some programs and resources exist, these are often limited and not always accessible to this population. School programs, social programs such as Supplemental Nutrition Assistance Program (SNAP), SNAP‐Ed, and Special Supplemental Nutrition Program for Women, Infants, and Children (WIC) were developed to improve nutrition and health outcomes in high‐risk populations and can all contribute to disease prevention [25].

The Healthy, Hunger‐Free Kids Act (HHFKA) of 2010 strengthened nutrition standards for meals and beverages provided through the National School Lunch, Breakfast, and Smart Snacks Programs. This policy affects 50 million children daily at 99,000 schools and has been linked to a decline in the risk of obesity in children living in poverty [68]. The American Heart Association explicitly states that improved school nutrition standards, such as those implemented under the HHFKA, have contributed to better nutrition security for children [69].

Opportunities also exist to leverage existing child health initiatives that are not yet focused on MASLD. For example, *Voices for Healthy Kids*—a joint initiative of the American Heart Association and the Robert Wood Johnson Foundation—has achieved more than 140 state and local policy successes in child health and could serve as a platform for MASLD‐focused advocacy [70]. Local bans on *SSBs* and ultraprocessed foods in schools, which have shown success in limited settings, could be expanded nationally to address dietary drivers of pediatric MASLD [71].

### 1.9. Nutrition Management in the Setting of Advanced Obesity Treatment for Pediatric MASLD

Lifestyle interventions and weight loss continue to be the cornerstone in the treatment of MASLD in children with obesity and overweight, which seems to be the most common phenotype of MASLD. Despite the benefits and effectiveness of lifestyle modifications, these interventions alone are often not sufficient to reach and maintain a healthy BMI and weight changes to provide sustained improvement in obesity‐related health outcomes including MASLD [72].

Studies have demonstrated the benefits of specific interventions and the effect of weight loss in improvement of transaminases, steatosis, and histology [73, 74].

Advance therapies for the management of MASLD in the setting of obesity including pharmacotherapy (e.g., glucagon‐like peptide‐1 receptor agonists [GLP‐1 RA], resmetirom, etc.), metabolic or endoscopic bariatric surgery, or a combination of therapies have become available for adult and pediatric patients and are recommended to be provided alongside nutrition and lifestyle changes [25, 75]. These new therapies, although effective, also come with a subset of risks that have a direct impact on nutrition, including prominent gastrointestinal side effects (e.g. gastroparesis, nausea, vomiting, and constipation), the risk of gallstones that can also cause pancreatitis, reduced hunger drive that can ultimately lead to inadequate caloric intake, dehydration, disordered eating, malnutrition, sarcopenia, osteopenia, mineral deficiencies, and an increased risk of fractures [76].

Nutrition modification is also key for the minimization of side effects and optimization of obesity therapies during obesity‐related MASLD treatment. Some interventions include providing frequent, small portions to minimize symptoms of gastroparesis; focusing on high‐protein and high‐fiber meals; development of optimal feeding routines to prevent undernutrition and disordered eating; use of nutritional supplements and multivitamins; and close monitoring of BMI, growth, body tissue distribution, and micronutrient deficiencies [77].

In children, the concern of abnormal development and impaired growth is always present, and the risks and benefits of weight loss medications and surgery, which also impact liver steatosis, should always be considered. Guidelines for pediatric obesity management advocate that advanced obesity therapies should be provided in the setting of pediatric multidisciplinary weight management programs guided by obesity medicine specialists to obtain optimal outcomes of obesity and obesity‐related heath outcome treatment, including MASLD [25, 78].

## 2. Conclusion

High‐calorie and low‐nutrient foods and beverages contribute significantly to the rising prevalence of MASLD. IHBLT alone has not been sufficient to curb this epidemic. Stigma, combined with peer and societal pressure to maintain a “healthy” weight, has contributed to the incidence of anxiety, mood, and EDs in children with MASLD. Assessing and addressing nutrient deficiencies, mood disturbances, and DEBs is critical to mitigating the long‐term consequences of this disease. Emphasizing, strategizing, and implementing nutrition management in the setting of advanced obesity treatment for pediatric MASLD, including pharmacotherapy such as GLP1‐RA and bariatric surgery, are primordial.

To effectively combat MASLD, targeted pharmacotherapies supported by multicenter pediatric trials are essential, along with policies that promote equitable food distribution, food security, and access to high‐quality nutrition and healthcare. Empowering patients, families, and communities through preventive and educational programs and creating healthier food environments at home, in schools, and across retail spaces are equally important. A comprehensive response would also include public awareness campaigns and strong advocacy for research. This includes support for NIH‐funded, pediatric investigator–initiated studies, as well as industry‐sponsored nutrition intervention trials (NITs) and randomized controlled trials (RCTs) that can advance evidence‐based treatments and preventive measures.

## Funding

No funding was received for this manuscript.

## Disclosure

**Literature review methodology:** A comprehensive literature search was conducted using databases including PubMed/MEDLINE and Embase. Keywords utilized in various combinations included the following: pediatric metabolic dysfunction‐associated steatotic liver disease (MASLD), metabolic dysfunction‐associated steatohepatitis (MASH), nutrition assessment, eating disorder, disordered eating, and food insecurities. The search was restricted to the most relevant articles published on the topic in English language.

The contents of this article do not necessarily represent the views or position of the American Association of the Study of Liver Disease, the United States Food and Drug Administration, the United States Department of Health and Human Services, or the United States Government.

## Conflicts of Interest

The authors declare no conflicts of interest.

## Data Availability

Data sharing is not applicable to this article as no datasets were generated or analyzed during the current study.
